# Prognostic impact and immunotherapeutic implications of NETosis‐related gene signature in gastric cancer patients

**DOI:** 10.1111/jcmm.18087

**Published:** 2023-12-26

**Authors:** Tian Xiang, Zhenhua Wei, Chen Ye, Gao Liu

**Affiliations:** ^1^ Department of Clinical Laboratory Center Central Hospital of Enshi Tujia and Miao Autonomous Prefecture Enshi China; ^2^ Hubei Minzu University Enshi China; ^3^ Hubei University of Medicine Shiyan China; ^4^ Department of Gastrointestinal Surgery Central Hospital of Enshi Tujia and Miao Autonomous Prefecture Enshi China

**Keywords:** gastric cancer, immune, immunotherapy, NETosis, prognosis

## Abstract

The role of NETosis and its related molecules remains unclear in gastric cancer. The data used in this study was directly downloaded from the Cancer Genome Atlas (TCGA) database. All analysis and plots are completed in R software using diverse R packages. In our study, we collected the list of NETosis‐related genes from previous publications. Based on the list and expression profile of gastric cancer patients from the TCGA database, we identified the NETosis‐related genes significantly correlated with patients survival. Then, CLEC6A, BST1 and TLR7 were identified through LASSO regression and multivariate Cox regression analysis for prognosis model construction. This prognosis model showed great predictive efficiency in both training and validation cohorts. We noticed that the high‐risk patients might have a worse survival performance. Next, we explored the biological enrichment difference between high‐ and low‐risk patients and found that many carcinogenic pathways were upregulated in the high‐risk patients. Meanwhile, we investigated the genomic instability, mutation burden and immune microenvironment difference between high‐ and low‐risk patients. Moreover, we noticed that low‐risk patients were more sensitive to immunotherapy (85.95% vs. 56.22%). High‐risk patients were more sensitive to some small molecules compounds like camptothecin_1003, cisplatin_1005, cytarabine_1006, nutlin‐3a (−)_1047, gemcitabine_1190, WZ4003_1614, selumetinib_1736 and mitoxantrone_1810. In summary, our study comprehensively explored the role of NETosis‐related genes in gastric cancer, which can provide direction for relevant studies.

## 1 INTRODUCTION

Gastric cancer (GC), remains one of the most challenging malignancies faced by the global health community.^1^ Originating from the inner lining of the stomach, GC can develop slowly over many years, often starting as precancerous changes.^2^ The symptoms of GC are often vague in the early stages, leading to late diagnosis in many cases, which can subsequently affect the prognosis.^3^, ^4^ Generally, GC is categorized into various types based on its cell type of origin, with adenocarcinoma being the most prevalent, accounting for about 90% of all cases.^5^ The factors contributing to GC are multifaceted, ranging from chronic gastritis, and Helicobacter pylori infection, to prolonged dietary and environmental exposures.^6^ Globally, it ranks as the fifth most diagnosed cancer and the third leading cause of cancer‐related deaths.^7^ Although the prevalence has been decreasing in many Western countries due to dietary improvements and better food preservation, it remains prevalent in Eastern Asia, Eastern Europe and South America.^8^ The early detection and understanding of its underlying molecular mechanisms are crucial for improving treatment strategies and patient outcomes.^9^

NETosis is an intriguing and unique form of programmed cell death, primarily exhibited by neutrophils, one of the frontline defenders in our immune system.^10^ Unlike traditional apoptosis or necrosis, during NETosis, neutrophils expel a web‐like structure composed of their DNA, histones, and various antimicrobial proteins.^11^ These expelled networks, termed Neutrophil Extracellular Traps (NETs), are primarily designed to ensnare and neutralize pathogens.^12^, ^13^ However, as with many physiological processes, there's a double‐edged sword to NETosis.^14^ In recent years, a growing body of research has started to unveil the multifaceted roles of NETosis, especially its implications in non‐infectious diseases.^15^ Among these, its association with cancer has generated significant interest. While on one hand, NETs might play a protective role by trapping circulating tumour cells, and preventing their metastasis, they have also been implicated in fostering tumour progression. This is attributed to the inflammatory and pro‐thrombotic nature of NETs, which can create a favourable microenvironment for tumour growth and spread.^16^ Moreover, components of NETs can also induce DNA damage in adjacent cells, potentially driving tumorigenesis.^17^ Furthermore, some studies suggest that the very presence of tumours can stimulate NETosis, thereby creating a feedback loop that can exacerbate disease progression.^18^ Understanding the intricate dance between NETosis and cancer holds promise not just in deciphering tumour biology, but also in potentially unveiling novel therapeutic targets that could revolutionize cancer management. As the dynamics of NETosis continue to be unravelled, its impact on cancer prognosis, diagnosis and therapy is anticipated to be profound.

## 2 METHODS

### 2.1 Data acquisition

Expression data of RNA sequencing and relevant clinical information of all STAD samples were downloaded from the Cancer Genome Atlas (TCGA) database (https://tcga‐data.nci.nih.gov/tcga/).^19^ After removing samples without clinical information or with less than 30 days of clinical follow‐up (Table 1). Then STAR‐Counts data was transformed into transcript per million (TPM) form and normalized through log2 (TPM + 1). Single‐cell sequencing data of STAD patients were downloaded from the TISCH database (http://tisch.comp‐genomics.org/home/). And 61 NETosis‐related genes (NRGs) were retrieved from previous publications.^12^, ^20^, ^21^

**TABLE 1:** The basic information of the enrolled patients.

Features	Subgroups	Counts	Percentage (%)
Age	<=65	197	44.5
	>65	241	54.4
	Unknown	5	1.1
Gender	Female	158	35.7
	Male	285	64.3
Grade	G1	12	2.7
	G2	159	35.9
	G3	263	59.4
	Unknown	9	2.0
Stage	Stage I	59	13.3
	Stage II	130	29.3
	Stage III	183	41.3
	Stage IV	44	9.9
	Unknown	27	6.1
T‐stage	T1	23	5.2
	T2	93	21.0
	T3	198	44.7
	T4	119	26.9
	Unknown	10	2.3
N‐stage	N0	132	29.8
	N1	119	26.9
	N2	85	19.2
	N3	88	19.9
	Unknown	19	4.3
M‐stage	M0	391	88.3
	M1	30	6.8
	Unknown	22	4.9

### 2.2 Construction and verification of prognostic signature based on NRGs

All the NRGs were included in the univariate Cox regression analysis, and the NRGs meaningful for univariate analysis were further incorporated into LASSO analysis and multivariate Cox regression analysis. The prognostic signature was established according to NRGs screened from multivariate Cox regression analysis.^22^ An external validation based on GSE84437cohort was performed to validate the predictive performance of the signature using multiple methods including differential clinical parameters analysis, Kaplan–Meier (KM) method analysis, time‐dependent receiver operating characteristic (ROC) and independent prognostic analysis.^22^

### 2.3 Functional analysis of the signature between two groups

With the ‘gsva’ R package, GSVA analysis was applied to the hallmark gene set to evaluate pathway enrichment.^23^ Then the latest KEGG pathway gene annotation was obtained from KEGG rest API (https://www.kegg.jp/kegg/rest/keggapi.html) for enrichment of gene set function analysis.^24^ GSE134520 and GSE167297 cohorts were downloaded from the online single‐cell portal database of the TISCH database (http://tisch.comp‐genomics.org/home/) for single‐cell data analysis.^25^, ^26^, ^27^

### 2.4 Exploration of phylogenomic features

Somatic mutation data used to calculate tumour mutational burden (TMB) were retrieved from from cBioPortal website (https://www.cbioportal.org/datasets).^28^ With maftools, we screened genes with *p*‐values lower than 0.05 that were different between the two risk groups and analysed how these mutations interact. In addition, copy number variation (CNV) analysis was applied using GISTIC_2.0 (https://cloud.genepattern.org). Copy number gain burden and loss burden at the focal and arm levels were calculated based on the output files from GISTIC_2.0.

### 2.5 Immune infiltration analysis

Single sample gene set enrichment analysis (ssGSEA) algorithm was implemented to estimate the scores of immune cells and immune functions between the two groups. The ESTIMATE algorithm was used to calculate the stromal scores, immune scores and ESTIMATE scores, which represented tumour immune score and immune cell infiltration status in two groups.

### 2.6 Immunotherapy and chemotherapy response prediction

The potential response to immune checkpoint blockade (ICB) of patients with STAD was assessed between high‐ and low‐risk groups based on the tumour immune dysfunction and exclusion (TIDE) (http://tide.dfci.harvard.edu).^29^, ^30^ SubMap (https://cloud.genepattern.org/gp) analysis was performed to estimate the immune checkpoint inhibitors response of PD‐1 and CTLA4 in two groups. The drug sensitivity to chemotherapy in STAD patients was estimated through the public database Genomics Drug Sensitivity in Cancer (GDSC).^31^

### 2.7 Statistical analysis

Statistical significance was determined by a *p*‐value of 0.05 for all two‐sided tests. A *t*‐test was performed on independent samples with continuous variables with a normal distribution to compare their means and standard deviations. For the comparison of continuous variables with skewed distributions, the Wilcoxon rank‐sum test was used.

## 3 RESULTS

### 3.1 Expression landscape of NRGs and signature construction

**FIGURE 1:**
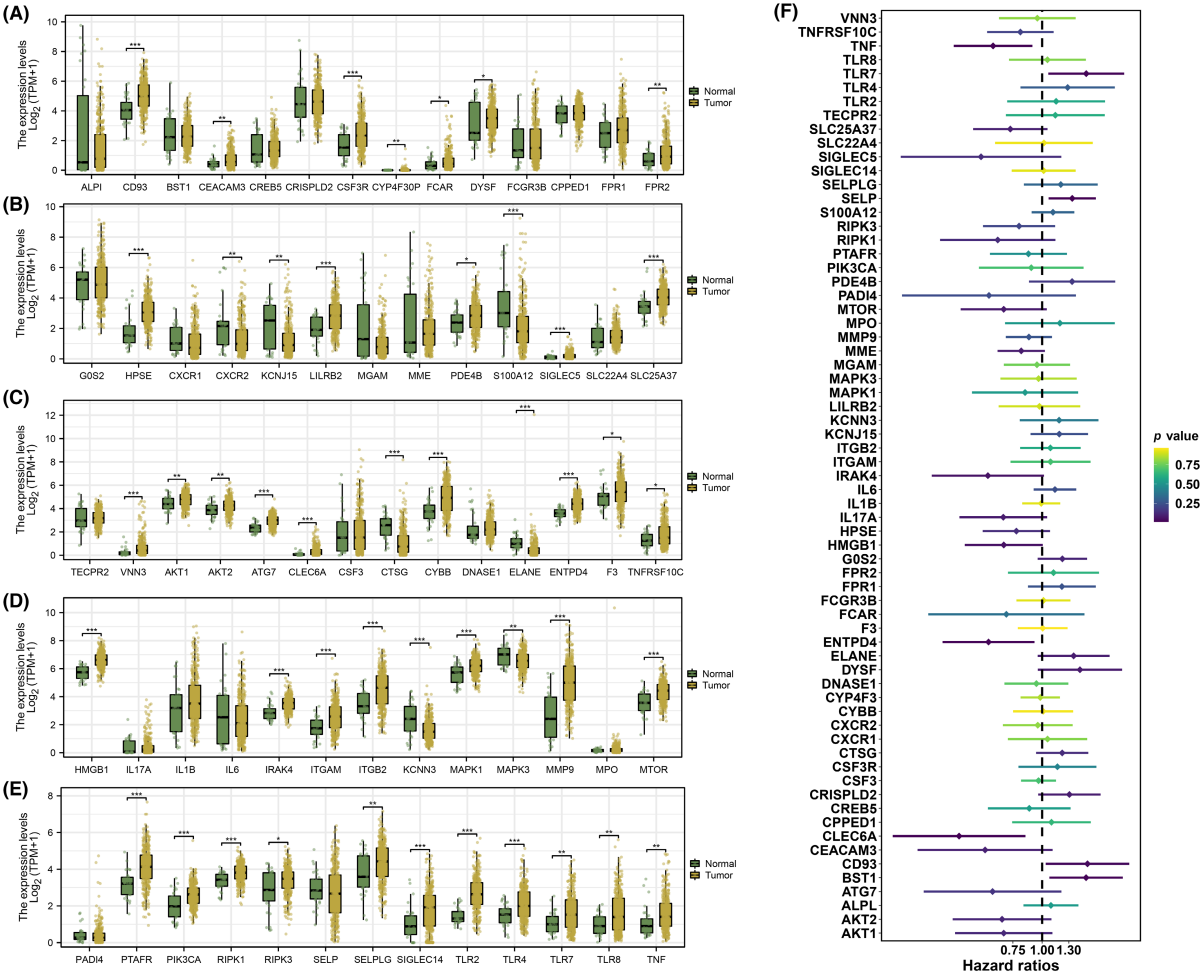
Expression of NETosis‐related genes (NRGs) and univariate prognostic analysis. (A–E) Results of differential expression analysis of all the NRGs between normal and STAD samples. (F) Results of univariate prognostic analysis of all the NRGs. * = *p* < 0.05; ** = *p* < 0.01; *** = *p* < 0.001; **** = *p* < 0.0001.

**FIGURE 2:**
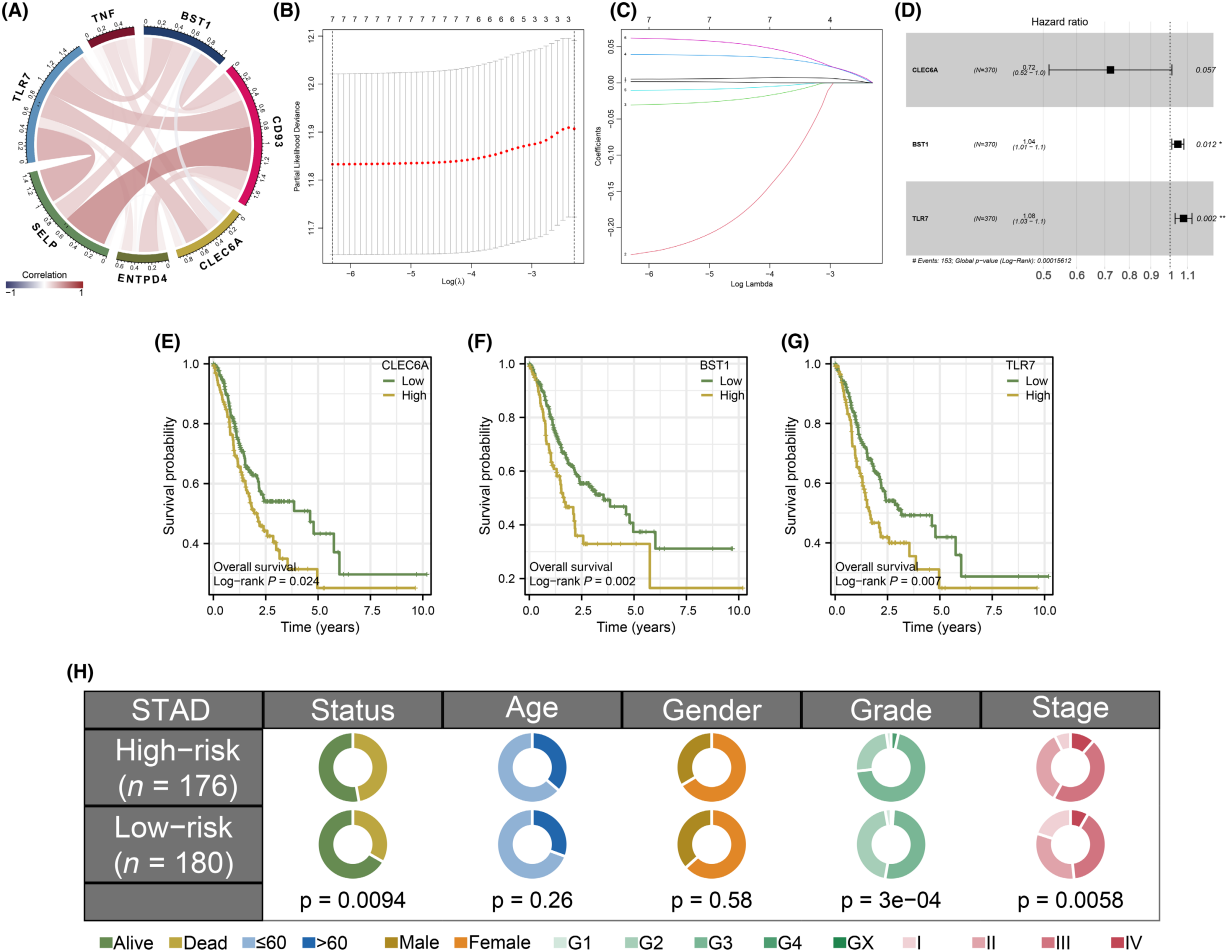
Identification of NETosis‐related genes (NRGs)‐based prognostic signature. (A) Correlation analysis between the NRGs screened from the univariate prognostic analysis. (B, C) LASSO regression analysis screened seven NRGs. (D) Forest plots of three prognostic NRGs identified by multivariate Cox regression analysis, (E–G) Kaplan–Meier survival curves of signature NRGs in STAD. (H) Pie charts showing the Chi‐squared test of clinicopathologic factors between two groups.

After the acquisition of all NRGs, differentially expressed NRGs were screened by differential expression analysis using ‘limma’ R packages (Figure 1A–E). The univariate Cox regression analysis was then performed and seven NRGs affecting prognosis were selected including TNF (ENSG00000232810), BST1 (ENSG00000109743), CD93 (ENSG00000125810), CLEC6A (ENSG00000205846), ENTPD4 (ENSG00000197217), SELP (ENSG00000174175) and TLR7 (ENSG00000196664) (Figure 1F). Figure 2B displays the correlations between these NRGs from univariate Cox regression analysis. To increase the predictive accuracy, we opted to additionally perform LASSO regression analysis and all these NRGs were selected for further analysis (Figure 2B, C). Following that, these NRGs were included to conduct multivariate Cox regression analysis, and a prognostic signature composed of three NRGs including CLEC6A, BST1 and TLR7 was successfully constructed with a riskScore formula: riskScore = the expression of CLEC6A* ‐0.325645123010234 + the expression of BST1* 0.0423937986799213 + the expression of TLR7* 0.0736051785940703 (Figure 2D). Then, STAD patients were stratified into a high‐risk group and a low‐risk group according to the median cutoff value. Based on these signature NRGs, Figure 2E–G illustrates the general probability of survival for patients based on Kaplan–Meier curves. And in clinical association analyses between riskScore and different clinicopathological features, we found that for STAD patients involved in a high‐risk group, the higher riskScore is related to the high grade and stage (Figure 2H).

### 3.2 Validation and evaluation of the signature

**FIGURE 3:**
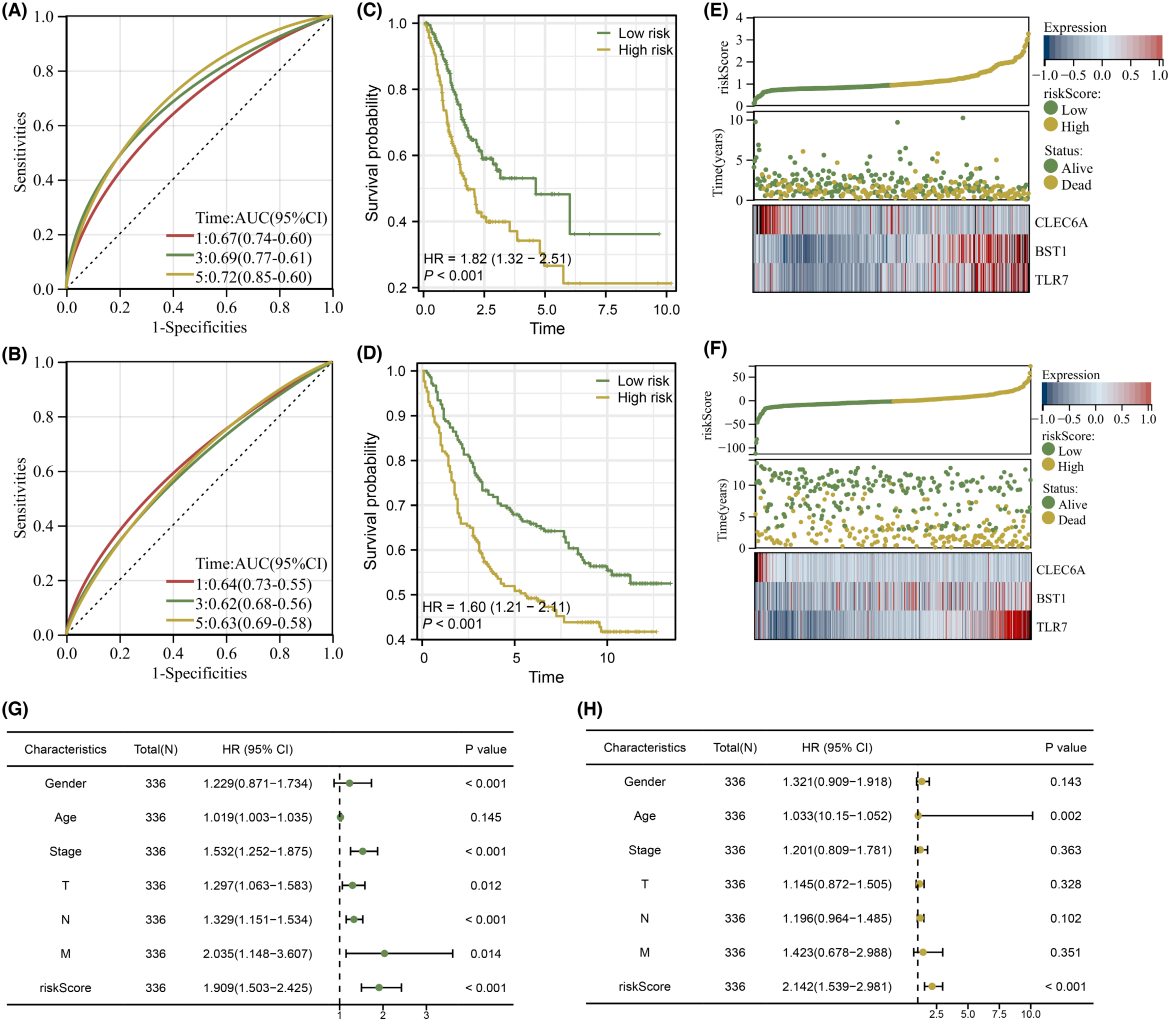
Verification and evaluation of the signature. (A, B) Both training and validation cohorts were evaluated based on AUC values under the time‐dependent receiver operating characteristic (ROC) curves. (C, D) Analysing the Kaplan–Meier survival curves in both the training and validation cohorts to compare overall survival between the two risk groups. (E, F) Distribution of survival status and survival time for patients with different risk scores as well as the expression levels of three prognostic NETosis‐related genes (NRGs) in both training and validation cohorts. (G, H) Prognostic value of riskScore measured by prognostic value of risk score measured by multivariate Cox regression analyses.

In addition to the STAD samples included from the TCGA database as the training cohort, an external validation cohort from GSE84437 was also obtained to validate the accuracy of the prognostic signature. Time‐dependent ROC curve analyses showed the overall accuracy was excellent among the both training and validation cohorts (Figure 3A, B). Also, KM survival curves showed that patients in the high‐risk group have a worse survival performance (Figure 3C, D). Meanwhile, from Figure 3E, F, we can see that higher levels of riskScore were associated with higher mortality risks for STAD patients in both training and validation cohorts. To verify whether the riskScore could act as an independent prognostic factor, we performed univariate and multivariate independent prognosis analyses, and the results showed the riskScore could serve as the independent prognostic factor with the lowest *p*‐value (Figure 3G, H).

### 3.3 Functional enrichment analysis and single‐cell analysis

**FIGURE 4:**
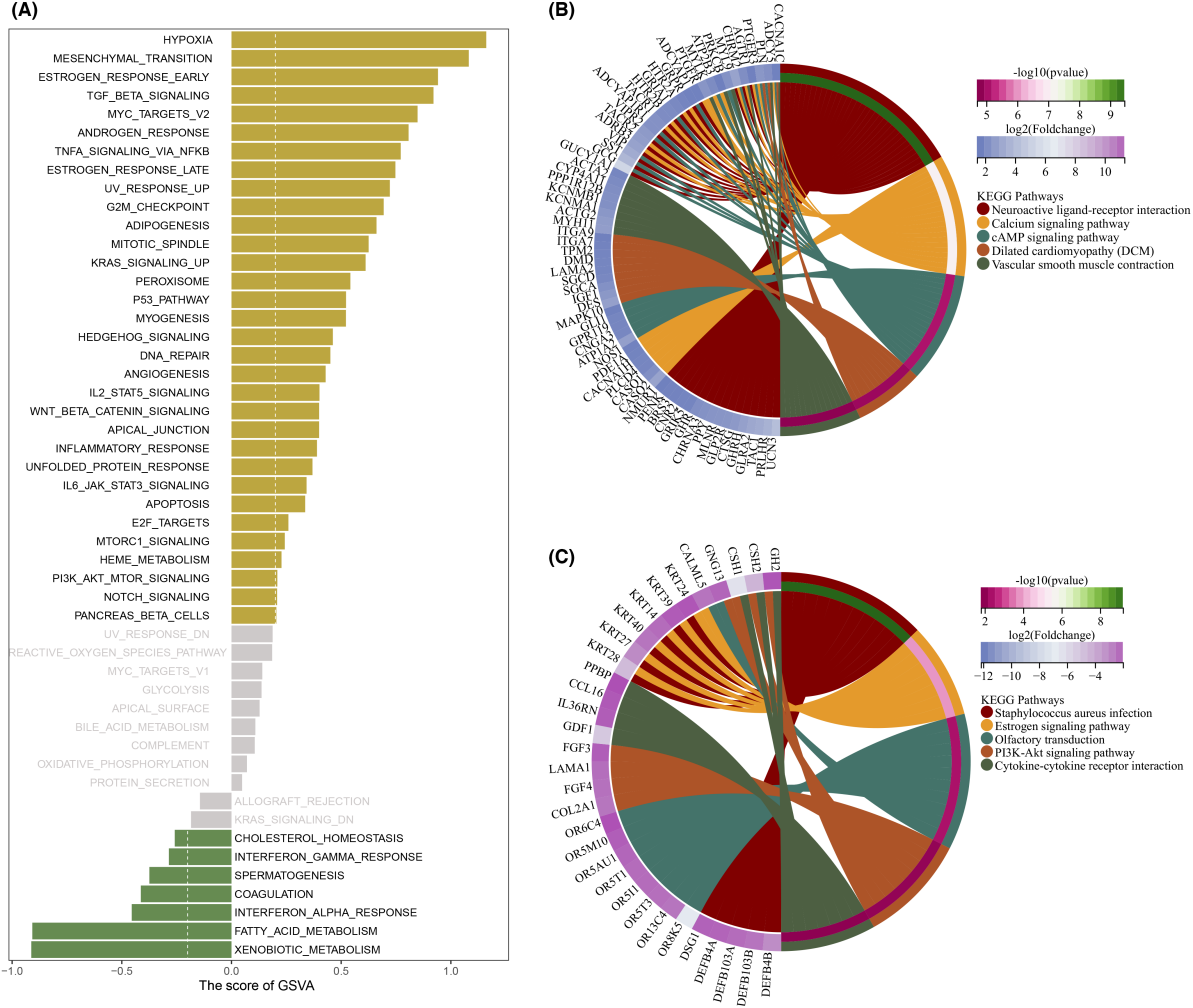
Analysis of single cells and functional enrichment. (A) GSVA analysis revealed two risk groups enriched in different functional pathways. (B, C) KEGG analysis showed the top five terms enriched in high‐risk and low‐risk groups.

In terms of the hallmark gene set as a reference gene set, GSVA was performed to investigate the potential biological functions and signalling pathways between the two groups. We found that in the high‐risk group, HYPOXIA, MESENCHYMAL_TRANSITION and OESTROGEN_RESPONSE_EARLY were enriched while XENOBIOTIC_METABOLISM, FATTY_ACID_METABOLISM and INTERFERON_ALPHA_RESPONSE were enriched in the low‐risk group (Figure 4A). Next, based on the top 100 differentially expressed genes selected from two risk groups, KEGG analysis was conducted, and the top five pathways in KEGG enrichment analysis were shown in both the high‐risk group (Figure 4B) and the low‐risk group (Figure 4C). Following this, the expression distribution of these NRGs in multiple cell subgroups was analysed both in the GSE134520 cohort and the GSE167297 cohort (Figure S1A, B).

### 3.4 Genomic features analysis including TMB and CNV

**FIGURE 5:**
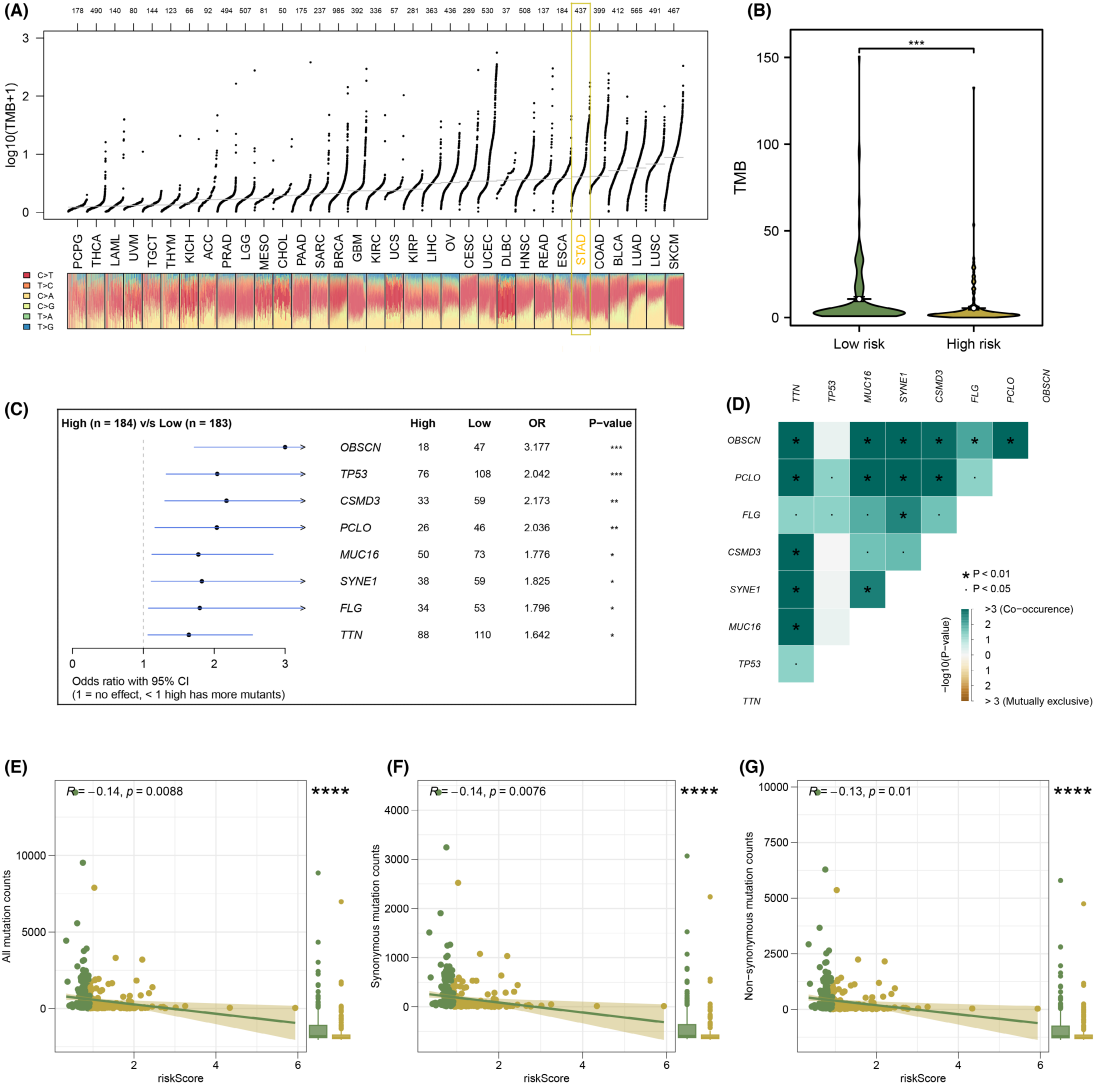
Correlation between risk score and tumour mutational burden (TMB). (A) TMB distribution of pan‐cancer in the Cancer Genome Atlas (TCGA). (B) Patients in the low‐risk group had a higher level of TMB. (C) A total of eight genes were identified mutated more frequently in the low‐risk group. (D) The heatmap showed the correlation between these eight genes identified by maftools analysis. (E–G) In patients with low riskScore, the number of somatic mutations, including non‐synonymous mutations, synonymous mutations and all mutations, was higher. * = *p* < 0.05; ** = *p* < 0.01; *** = *p* < 0.001; **** = *p* < 0.0001.

**FIGURE 6:**
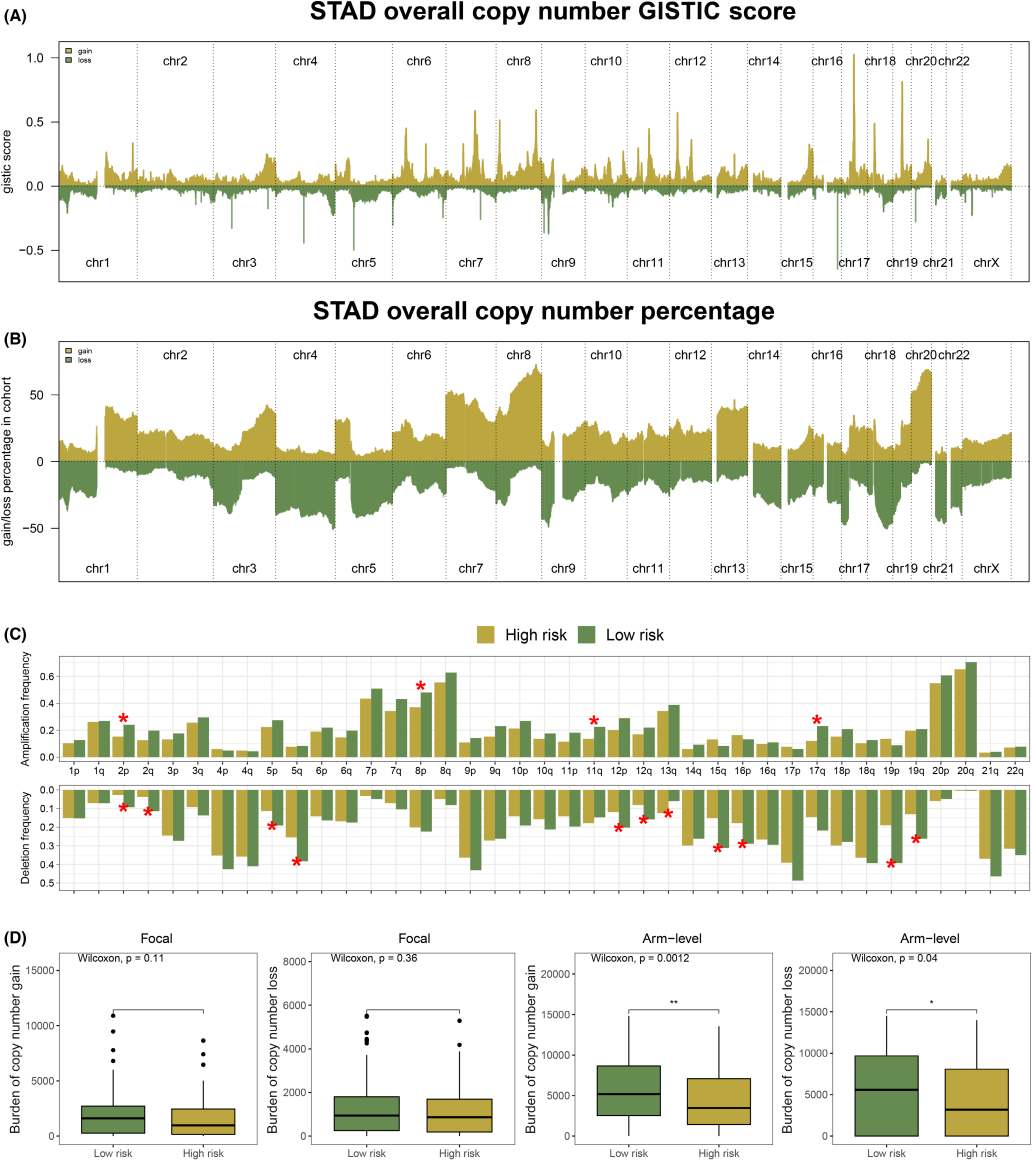
Copy number variation (CNV) analysis between two groups. (A) Overall copy number GISTIC score of each STAD patient. (B) Overall copy number percentage of each STAD patient. (C) Between different risk groups, there was a significant difference in amplification and deletion frequencies. (D) Patients with low riskScore had a higher level of burden of number gain or loss at arm level. * = *p* < 0.05; ** = *p* < 0.01; *** = *p* < 0.001; **** = *p* < 0.0001.

Based on analysis of the TCGA database, we found that STAD patients had a higher TMB level than patients with other types of cancers (Figure 5A). Patients in the low‐risk group had a lower level of TMB score than patients in the high‐risk group (Figure 5B). Meanwhile, to identify differences in mutated genes between high‐ and low‐risk groups, analysis of differentiation of mutated genes was performed, and we found that almost all genes were prone to be more susceptible to mutation in the low‐risk group (Figure 5C). Then, somatic mutations data of STAD samples were also analysed, and we found more somatic mutations including non‐synonymous, synonymous mutations and all mutations enriched in patients with low‐risk (Figure 5E–G). Meanwhile, the interrelationships of these genes are also shown in Figure 5D. For each patient with STAD and GISTIC scores were calculated to compare CNV between the two risk groups (Figure 6A, B). Further, based on the differential CNV analysis, we found that STAD patients in the high‐risk group possessed lower CNV losses or gains at arm level than STAD patients in the low‐risk group (Figure 6C, D).

### 3.5 Multiple immune analyses revealed the correlations between riskScore and the immunotherapy response

**FIGURE 7:**
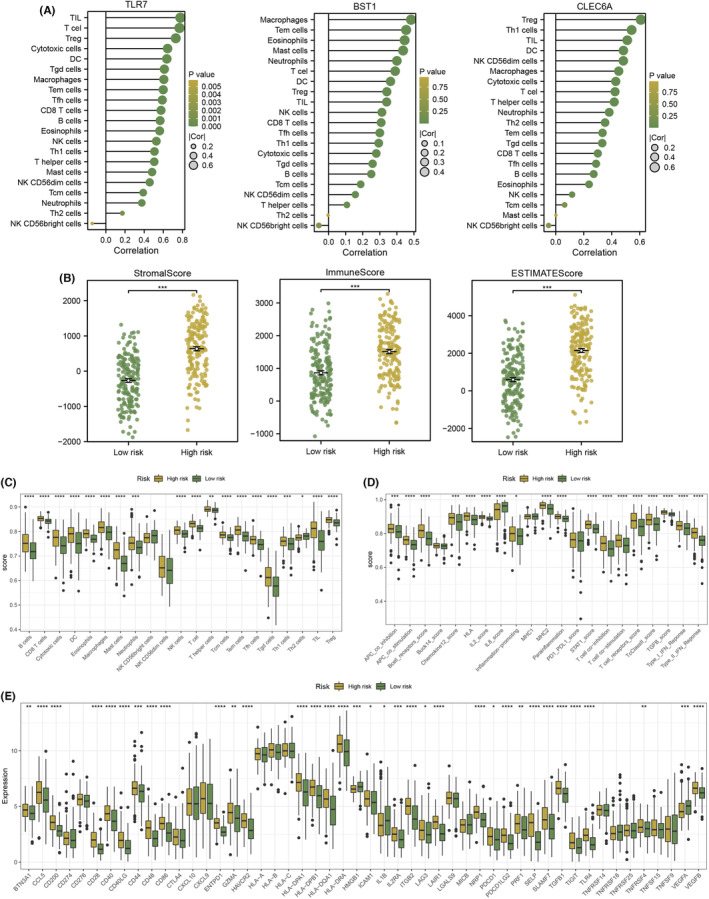
The immune infiltration landscape between two risk groups. (A) The relationship between immune cells scores and three hub NETosis‐related genes (NRGs). (B) The comparison of the stromal scores, immune scores and ESTIMATE scores between high‐ and low‐risk groups. (C, D) The immunity infiltration differences between the two groups. (E) The difference in expression of immune checkpoint between two groups. * = *p* < 0.05; ** = *p* < 0.01; *** = *p* < 0.001; **** = *p* < 0.0001.

**FIGURE 8:**
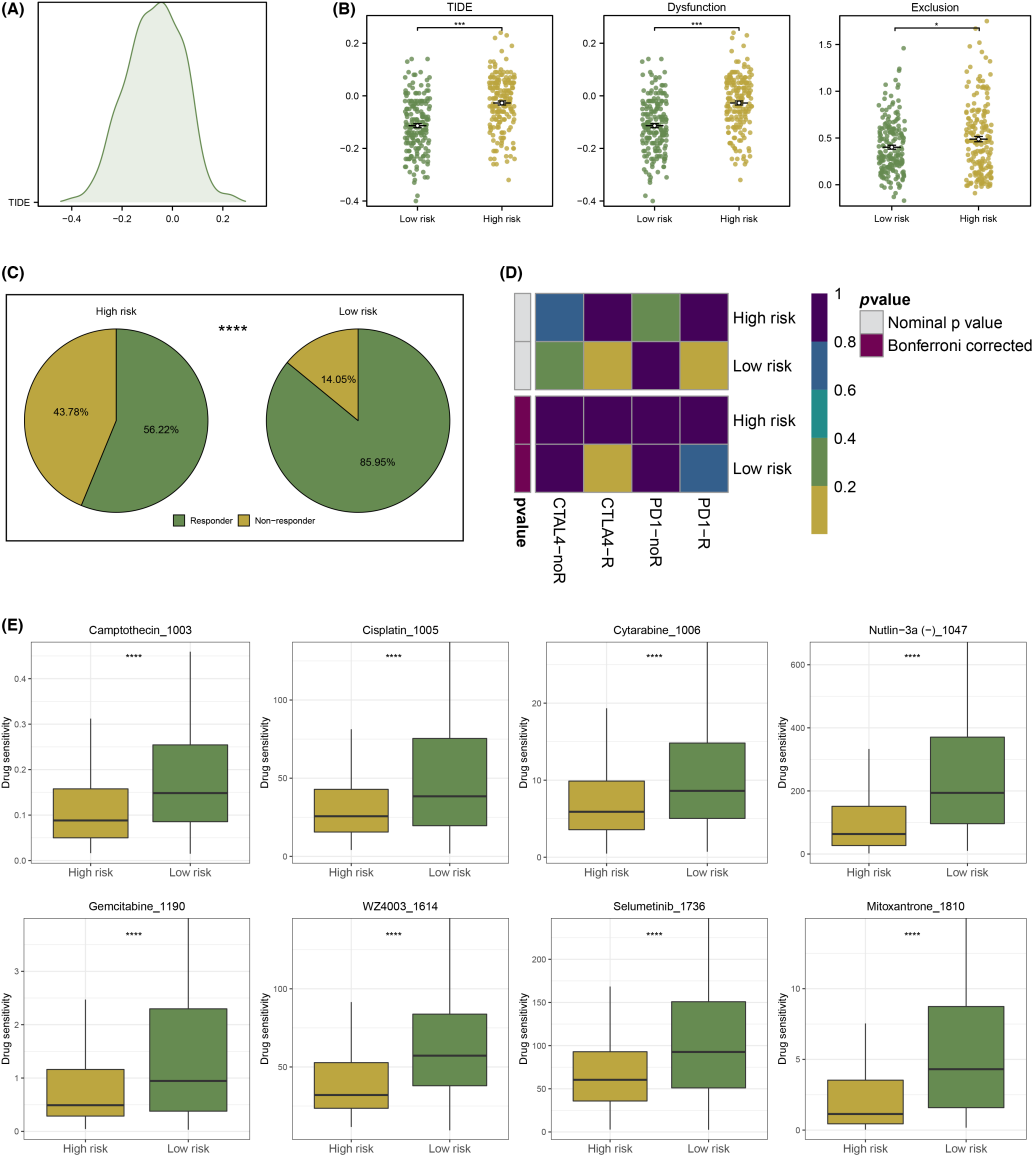
Prediction of immunotherapy response and chemotherapy sensitivity. (A) The distribution of TIDE scores in patients with STAD. (B) The comparison of TIDE, dysfunction and exclusion scores between high‐ and low‐risk groups. (C) The proportion of responders to immunotherapy in two groups according to the TIDE algorithm. (D) Response prediction to immunotherapy between high‐ and low‐risk groups according to the SubMap algorithm. (E) Response prediction to chemotherapy between two groups.

We explored the relationship between immune cell scores and three hub NRGs and found that there was a strong positive correlation among them (Figure 7A). Besides, the stromal scores, immune scores and ESTIMATE scores were significantly lower in the low‐risk group than in the high‐risk group (Figure 7B). The most of immune cell scores and immune function scores including B cells, T cells, APC co‐inhibition, and type I IFN response were significantly lower in the low‐risk group than in the high‐risk group (Figure 7C, D). The expression of immune checkpoints like PDCD1 and TIGIT was also higher in the high‐risk group than in the low‐risk group (Figure 7E). Furthermore, the distribution of TIDE scores in STAD patients was exhibited (Figure 8A). Compared to the high‐risk group, the TIDE, dysfunction and exclusion scores were significantly lower in the low‐risk group (Figure 8B). Patients in the low‐risk group had a higher proportion of responders to immunotherapy than high‐risk group (Figure 8C). The result of the SubMap analysis also revealed that patients in the low‐risk group may respond to anti‐CTLA4 immunotherapy than in the high‐risk group (Figure 8D). In addition, we found that Camptothecin_1003, Cisplatin_1005, Cytarabine_1006, Nutlin−3a (−)_1047, Gemcitabine_1190, WZ4003_1614, Selumetinib_1736, and Mitoxantrone_1810 witnessed significant drug sensitivity differences between high‐ and low‐risk group (Figure 8E).

## 4 DISCUSSION

GC, often viewed as a significant public health concern, maintains a consistently high prevalence globally.^32^ This malignancy subtly initiates from the inner lining cells of the stomach and progressively develops.^33^ Many patients, in the early stages, remain asymptomatic, leading to frequent late‐stage diagnoses.^34^ When persistent symptoms such as upper abdominal pain or indigestion emerge, the disease may have already advanced to a critical phase. Lifestyle choices, dietary habits and familial genetics can all elevate the risk of contracting this ailment. Confronted with such a health menace, the pursuit of superior preventive and therapeutic approaches becomes an urgent endeavour in the medical community.

In our study, we collected the list of NETosis‐related genes from previous publications. Based on the list and expression profile of GC patients from the TCGA database, we identified the NETosis‐related genes significantly correlated with patients survival. Then, CLEC6A, BST1 and TLR7 were identified through LASSO regression and multivariate Cox regression analysis for prognosis model construction. This prognosis model showed great predictive efficiency in both training and validation cohorts. We noticed that the high‐risk patients might have a worse survival performance. Next, we explored the biological enrichment difference between high‐ and low‐risk patients and found that many carcinogenic pathways were upregulated in the high‐risk patients. Meanwhile, we investigated the genomic instability, mutation burden and immune microenvironment difference between high‐ and low‐risk patients. Moreover, we noticed that low‐risk patients were more sensitive to immunotherapy (85.95% vs. 56.22%). High‐risk patients were more sensitive to some small molecules compounds like camptothecin_1003, cisplatin_1005, cytarabine_1006, nutlin‐3a (−)_1047, gemcitabine_1190, WZ4003_1614, selumetinib_1736 and mitoxantrone_1810.

Distinctive pathway enrichments were observed between high‐risk and low‐risk groups. Notably, in the high‐risk group, pathways such as HYPOXIA, MESENCHYMAL_TRANSITION and OESTROGEN_RESPONSE_EARLY were prominently enriched. Hypoxia is a well‐documented factor in tumour progression, often contributing to enhanced tumour cell survival, migration and invasion.^35^ Concurrently, mesenchymal transition, a hallmark of epithelial‐mesenchymal transition, is frequently associated with tumour metastasis and resistance to therapies, further highlighting its relevance in a high‐risk patients.^36^ The enrichment of the early oestrogen response might suggest a hormonal influence in the progression or maintenance of the high‐risk phenotype, hinting at potential therapeutic targets.^37^ Conversely, the low‐risk group displayed enrichment in pathways such as XENOBIOTIC_METABOLISM, FATTY_ACID_METABOLISM and INTERFERON_ALPHA_RESPONSE. Xenobiotic metabolism pathways play a crucial role in the detoxification of foreign substances, suggesting that the low‐risk group might possess a heightened capability to metabolize potentially harmful compounds. The prominence of fatty acid metabolism indicates potential differences in energy utilization or lipid synthesis between the two groups. Meanwhile, the enrichment of interferon‐alpha response is particularly intriguing.^38^ Interferons, especially alpha‐type, are renowned for their antiviral and antitumor properties. Their upregulation in the low‐risk group might reflect an innate capacity to mount a more robust defence against tumour progression or external pathogens. Taken together, these findings illuminate the complex molecular landscape that differentiates high‐risk from low‐risk groups. Understanding these nuances not only enhances our comprehension of disease pathogenesis but also paves the way for targeted therapeutic strategies.

Meanwhile, a compelling observation was the stark difference in immune cell scores and immune function scores between the low‐risk and high‐risk groups. The majority of these scores, including those for B cells, T cells, APC co‐inhibition and type I IFN response, were notably diminished in the low‐risk group compared to the high‐risk group. The lower scores for B cells and T cells in the low‐risk group suggest a potential reduction in adaptive immune responses.^39^ This is intriguing, as one might typically associate robust adaptive immune responses with effective antitumor defences. However, it's possible that in this context, a heightened adaptive immune response may reflect or contribute to an inflammatory microenvironment, which has been implicated in promoting tumour progression and metastasis in numerous malignancies.^40^ Furthermore, the decreased scores for APC co‐inhibition in the low‐risk group might indicate reduced levels of immune checkpoint molecules, which are often hijacked by tumours to evade immune surveillance. This could mean that the low‐risk group has a diminished requirement for co‐inhibitory signalling, possibly due to a more quiescent immune landscape. Lastly, the attenuated type I IFN response in the low‐risk group could reflect reduced antiviral or antitumor activities. Type I interferons are crucial players in innate immunity, often acting as the first line of defence against pathogens and malignancies. Their reduced activity could suggest a more general suppression of innate immune responses in the low‐risk group. In sum, our findings underscore the intricacies of the immune landscape in the context of disease risk. A deeper understanding of these immunological differences might reveal pivotal insights into disease progression mechanisms and offer innovative avenues for therapeutic interventions.

## AUTHOR CONTRIBUTIONS

**Tian Xiang:** Formal analysis (equal); funding acquisition (equal); investigation (equal); methodology (equal); resources (equal); software (equal); supervision (equal); validation (equal). **Zhenhua Wei:** Data curation (equal); investigation (equal); methodology (equal); resources (equal); supervision (equal); writing – original draft (equal). **Chen Ye:** Formal analysis (equal); funding acquisition (equal); investigation (equal); supervision (equal); validation (equal); visualization (equal). **Gao Liu:** Conceptualization (equal); data curation (equal); formal analysis (equal); methodology (equal); supervision (equal); validation (equal).

## FUNDING INFORMATION

This work was supported by the National Natural Science Foundation of China (82360477 and 82060539); the Natural Science Foundation of Hubei Province of China (2022CFB344), and the Scientific and Technological Project of Enshi Tujia and Miao Autonomous Prefecture of Hubei Province (D20220059).

## CONFLICT OF INTEREST STATEMENT

None.

## Supporting information


Figure S1.

## Data Availability

All data are available from the corresponding author upon reasonable request.

## References

[1] Oliveira C , Pinheiro H , Figueiredo J , Seruca R , Carneiro F . Familial gastric cancer: genetic susceptibility, pathology, and implications for management. Lancet Oncol. 2015;16(2):e60‐e70. doi:10.1016/s1470-2045(14)71016-2 25638682

[2] Catalano V , Labianca R , Beretta GD , Gatta G , de Braud F , Van Cutsem E . Gastric cancer. Crit Rev Oncol Hematol. 2005;54(3):209‐241. doi:10.1016/j.critrevonc.2005.01.002 15890270

[3] Smyth EC , Nilsson M , Grabsch HI , van Grieken NC , Lordick F . Gastric cancer. Lancet. 2020;396(10251):635‐648. doi:10.1016/s0140-6736(20)31288-5 32861308

[4] Karimi P , Islami F , Anandasabapathy S , Freedman ND , Kamangar F . Gastric cancer: descriptive epidemiology, risk factors, screening, and prevention. Cancer Epidemiol Biomarkers Prev. 2014;23(5):700‐713. doi:10.1158/1055-9965.Epi-13-1057 24618998 PMC4019373

[5] Hohenberger P , Gretschel S . Gastric cancer. Lancet. 2003;362(9380):305‐315. doi:10.1016/s0140-6736(03)13975-x 12892963

[6] Venerito M , Link A , Rokkas T , Malfertheiner P . Gastric cancer–clinical and epidemiological aspects. Helicobacter. 2016;21(Suppl 1):39‐44. doi:10.1111/hel.12339 27531538

[7] Sexton RE , Hallak MNA , Uddin MH , Diab M , Azmi AS . Gastric cancer heterogeneity and clinical outcomes. Technol Cancer Res Treat. 2020;19:1533033820935477. doi:10.1177/1533033820935477 32799763 PMC7432987

[8] Chiarello MM , Fico V , Pepe G , et al. Early gastric cancer: a challenge in Western countries. World J Gastroenterol. 2022;28(7):693‐703. doi:10.3748/wjg.v28.i7.693 35317273 PMC8891729

[9] Leja M , Linē A . Early detection of gastric cancer beyond endoscopy–new methods. Best Pract Res Clin Gastroenterol. 2021;50‐51:101731. doi:10.1016/j.bpg.2021.101731 33975677

[10] Thiam HR , Wong SL , Wagner DD , Waterman CM . Cellular mechanisms of NETosis. Annu Rev Cell Dev Biol. 2020;36:191‐218. doi:10.1146/annurev-cellbio-020520-111016 32663035 PMC8499668

[11] Vorobjeva NV , Chernyak BV . NETosis: molecular mechanisms, role in physiology and pathology. Biochemistry (Mosc). 2020;85(10):1178‐1190. doi:10.1134/s0006297920100065 33202203 PMC7590568

[12] Papayannopoulos V . Neutrophil extracellular traps in immunity and disease. Nat Rev Immunol. 2018;18(2):134‐147. doi:10.1038/nri.2017.105 28990587

[13] Masucci MT , Minopoli M , Del Vecchio S , Carriero MV . The emerging role of neutrophil extracellular traps (NETs) in tumor progression and metastasis. Front Immunol. 2020;11:1749. doi:10.3389/fimmu.2020.01749 33042107 PMC7524869

[14] Chen T , Li Y , Sun R , et al. Receptor‐mediated NETosis on neutrophils. Front Immunol. 2021;12:775267. doi:10.3389/fimmu.2021.775267 34804066 PMC8600110

[15] Hu Y , Wang H , Liu Y . NETosis: sculpting tumor metastasis and immunotherapy. Immunol Rev. 2023. doi:10.1111/imr.13277 37712361

[16] Cedervall J , Hamidi A , Olsson AK . Platelets, NETs and cancer. Thromb Res. 2018;164(Suppl 1):S148‐S152. doi:10.1016/j.thromres.2018.01.049 29703474

[17] Cristinziano L , Modestino L , Antonelli A , et al. Neutrophil extracellular traps in cancer. Semin Cancer Biol. 2022;79:91‐104. doi:10.1016/j.semcancer.2021.07.011 34280576

[18] Yang L , Liu Q , Zhang X , et al. DNA of neutrophil extracellular traps promotes cancer metastasis via CCDC25. Nature. 2020;583(7814):133‐138. doi:10.1038/s41586-020-2394-6 32528174

[19] Tomczak K , Czerwińska P , Wiznerowicz M . The cancer genome atlas (TCGA): an immeasurable source of knowledge. Contemp Oncol (Pozn). 2015;19(1a):A68‐A77. doi:10.5114/wo.2014.47136 25691825 PMC4322527

[20] Qi L , Chen F , Wang L , Yang Z , Zhang W , Li Z . Deciphering the role of NETosis‐related signatures in the prognosis and immunotherapy of soft‐tissue sarcoma using machine learning. Front Pharmacol. 2023;14:1217488. doi:10.3389/fphar.2023.1217488 37408763 PMC10318157

[21] Zhang Y , Guo L , Dai Q , et al. A signature for pan‐cancer prognosis based on neutrophil extracellular traps. J Immunother Cancer. 2022;10(6):e004210. doi:10.1136/jitc-2021-004210 35688556 PMC9189842

[22] Abd ElHafeez S , Torino C , D'Arrigo G , et al. An overview on standard statistical methods for assessing exposure‐outcome link in survival analysis (Part II): the Kaplan–Meier analysis and the cox regression method. Aging Clin Exp Res. 2012;24(3):203‐206. doi:10.1007/bf03325249 23114547

[23] Hänzelmann S , Castelo R , Guinney J . GSVA: gene set variation analysis for microarray and RNA‐seq data. BMC Bioinformatics. 2013;14:7. doi:10.1186/1471-2105-14-7 23323831 PMC3618321

[24] Kanehisa M , Goto S . KEGG: kyoto encyclopedia of genes and genomes. Nucleic Acids Res. 2000;28(1):27‐30. doi:10.1093/nar/28.1.27 10592173 PMC102409

[25] Zhang P , Yang M , Zhang Y , et al. Dissecting the single‐cell transcriptome network underlying gastric premalignant lesions and early gastric cancer. Cell Rep. 2019;27(6):1934‐1947.e5. doi:10.1016/j.celrep.2019.04.052 31067475

[26] Zhang M , Feng C , Zhang X , et al. Susceptibility factors of stomach for Sars‐Cov‐2 and treatment implication of mucosal protective agent in Covid‐19. Front Med. 2020;7:597967. doi:10.3389/fmed.2020.597967 PMC7840564 33521016

[27] Jeong HY , Ham IH , Lee SH , et al. Spatially distinct reprogramming of the tumor microenvironment based on tumor invasion in diffuse‐type gastric cancers. Clin Cancer Res. 2021;27(23):6529‐6542. doi:10.1158/1078-0432.Ccr-21-0792 34385296

[28] Gao J , Aksoy BA , Dogrusoz U , et al. Integrative analysis of complex cancer genomics and clinical profiles using the cBioPortal. Sci Signal. 2013;6(269):pl1. doi:10.1126/scisignal.2004088 23550210 PMC4160307

[29] Fu J , Li K , Zhang W , et al. Large‐scale public data reuse to model immunotherapy response and resistance. Genome Med. 2020;12(1):21. doi:10.1186/s13073-020-0721-z 32102694 PMC7045518

[30] Jiang P , Gu S , Pan D , et al. Signatures of T cell dysfunction and exclusion predict cancer immunotherapy response. Nat Med. 2018;24(10):1550‐1558. doi:10.1038/s41591-018-0136-1 30127393 PMC6487502

[31] Yang W , Soares J , Greninger P , et al. Genomics of drug sensitivity in cancer (GDSC): a resource for therapeutic biomarker discovery in cancer cells. Nucleic Acids Res. 2013;41(Database issue):D955‐D961. doi:10.1093/nar/gks1111 23180760 PMC3531057

[32] Song Z , Wu Y , Yang J , Yang D , Fang X . Progress in the treatment of advanced gastric cancer. Tumour Biol. 2017;39(7):1010428317714626. doi:10.1177/1010428317714626 28671042

[33] Hata H , Hiraoka N , Ojima H , Shimada K , Kosuge T , Shimoda T . Carcinoid tumor arising in a duplication cyst of the duodenum. Pathol Int. 2006;56(5):272‐278. doi:10.1111/j.1440-1827.2006.01957.x 16669876

[34] Ang TL , Fock KM . Clinical epidemiology of gastric cancer. Singapore Med J. 2014;55(12):621‐628. doi:10.11622/smedj.2014174 25630323 PMC4291998

[35] Mennerich D , Kubaichuk K , Kietzmann T . DUBs, Hypoxia, and cancer. Trends Cancer. 2019;5(10):632‐653. doi:10.1016/j.trecan.2019.08.005 31706510

[36] Pastushenko I , Blanpain C . EMT transition states during tumor progression and metastasis. Trends Cell Biol. 2019;29(3):212‐226. doi:10.1016/j.tcb.2018.12.001 30594349

[37] Liang J , Shang Y . Estrogen and cancer. Annu Rev Physiol. 2013;75:225‐240. doi:10.1146/annurev-physiol-030212-183708 23043248

[38] Borden EC . Interferons Α and Β in cancer: therapeutic opportunities from new insights. Nat Rev Drug Discov. 2019;18(3):219‐234. doi:10.1038/s41573-018-0011-2 30679806

[39] Horii M , Matsushita T . Regulatory B cells and T cell regulation in cancer. J Mol Biol. 2021;433(1):166685. doi:10.1016/j.jmb.2020.10.019 33096106

[40] Vesely MD , Kershaw MH , Schreiber RD , Smyth MJ . Natural innate and adaptive immunity to cancer. Annu Rev Immunol. 2011;29:235‐271. doi:10.1146/annurev-immunol-031210-101324 21219185

