# Supplementary figures and images for: Prognostic impact and immunotherapeutic implications of NETosis‐related gene signature in gastric cancer patients
Source: J Cell Mol Med. 2023 Dec 26;28(5):e18087. doi: 10.1111/jcmm.18087 (PMC10902305; doi:10.1111/jcmm.18087)

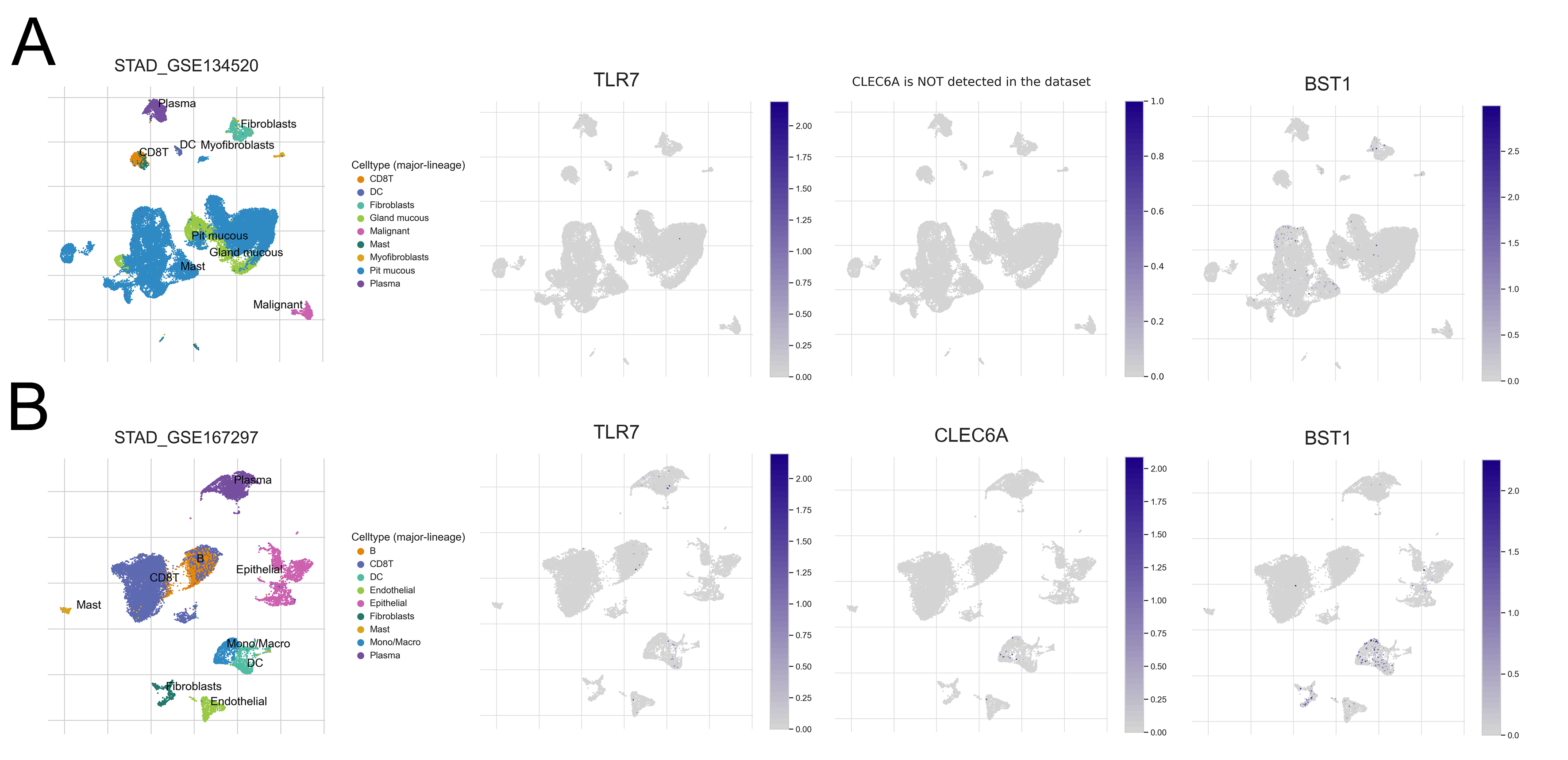

Supplement: Supplementary file 1 — Figure S1. [file JCMM-28-e18087-s001.tif]
